# Participatory Design of a Web-Based HIV Oral Self-Testing Infographic Experiment (HOTIE) for Emerging Adult Sexual Minority Men of Color: A Mixed Methods Randomized Control Trial

**DOI:** 10.3390/ijerph182211881

**Published:** 2021-11-12

**Authors:** S. Raquel Ramos, David T. Lardier, Keosha T. Bond, Donte T. Boyd, Olivia M. O’Hare, LaRon E. Nelson, Barbara J. Guthrie, Trace Kershaw

**Affiliations:** 1 School of Nursing, Yale University, Orange, CT 06477, USA; laron.nelson@yale.edu; 2 Department of Psychiatry and Behavioral Sciences, School of Medicine, University of New Mexico, Albuquerque, NM 87131, USA; dalardier@salud.unm.edu; 3 School of Medicine, The City University of New York, New York, NY 10031, USA; kbond@med.cuny.edu; 4 College of Social Work, The Ohio State University, Columbus, OH 43210, USA; boyd.465@osu.edu; 5 College of Nursing, New York University, New York, NY 10010, USA; ooh205@nyu.edu; 6 School of Public Health, Yale University, New Haven, CT 06520, USA; trace.kershaw@yale.edu; 7 Center for Interdisciplinary Research on AIDS, Yale University, New Haven, CT 06520, USA; 8 School of Nursing, Bouvé College of Health Sciences, Northeastern University, Boston, MA 02115, USA; barbara.guthrie@yale.edu

**Keywords:** consumer health informatics, health literacy, health communication, HIV, sexual and gender minorities, participatory design

## Abstract

Health communication is a key health promotion approach for translating research findings into actionable information. The purpose of this study was to use participatory design to create and then test the usability and comprehension of an HIV self-testing infographic in a sample of 322 emerging adult, sexual minority men of color. Our study objectives addressed three challenges to HIV self-testing: (1) correct usage of the test stick, (2) understanding the number of minutes to wait before reading the result, and (3) how to correctly interpret a negative or a positive HIV result. This study was a two-phase, sequential, mixed methods, pilot, online, randomized controlled trial. Results suggested a significant mean difference between the control and intervention groups on HIV self-testing knowledge, with the control group outperforming the intervention group. However, two-thirds or better of the participants in the intervention group were able to comprehend the three critical steps to HIV self-testing. This was a promising finding that has resulted in the authors’ development of additional recommendations for using participatory design for visual aid development in HIV prevention research. Participatory design of an HIV self-testing infographic is a rigorous approach, as a health communication strategy, to address public health priorities.

## 1. Introduction

Health communication is a key health promotion approach for translating research findings into actionable information [1]. This can be an especially meaningful strategy in populations that are marginalized due to systemic racism, discrimination, limited access to reliable sources of health information, language and/or literacy barriers, or other environmental factors [1]. A research strategy that has been found to strongly support comprehension and literacy outcomes, associated with health communication interventions, is participatory design [1]. Participatory design is based on a traditional constructivist paradigm [2] that fosters research collaborations with experts and potential users for the design of a product or tool that is tailored to the intended users [3]. A main tenet of participatory design is the early and ongoing involvement of user representatives in the design process [4]. Because of this, it has become critically necessary for researchers to consider how they can leverage participatory design approaches to develop chronic illness prevention interventions that are educational and also actionable.

Despite robust prevention initiatives at the national, state, and local levels, human immunodeficiency virus (HIV) remains a critical health issue in gay, queer, and bisexual-identified men (herein termed sexual minority men). In 2019, there were close to 37 thousand new HIV diagnoses [5]. In 2019, the Centers for Disease Control and Prevention (CDC) reported that an estimated 69% of newly diagnosed HIV, in the United States, were contracted by male sexual minority emerging adults ages 25–34 [5]. The Joint United National Programme on HIV/AIDS (UNAIDS) created targets for HIV treatment scale-up, referred to as the 90-90-90 strategy [6]. One of the targets is early identification of HIV through expanded testing. According to the 90-90-90 initiative, 90% of all people living with HIV will be serostatus aware via increased HIV testing strategies; those diagnosed with HIV will be initiated on antiretroviral therapy and will subsequently achieve viral load suppression [6]. Specifically, it was proposed that early identification can be achieved by increasing the number of persons who are made aware of their HIV serostatus [7]. HIV self-testing is one modality that can be employed to achieve this goal.

On 3 July 2012, the US Food and Drug Administration (FDA) approved the use of an over-the-counter, in-home, oral, HIV self-test [8]. The HIV oral self-test works by detecting the presence of HIV-1 and HIV-2 antibodies in oral fluid. Clinical studies of the oral HIV self-test have demonstrated that the test has 99.9% specificity and 91.7% sensitivity [8,9]. The sensitivity rate of the oral self-test is approximately 2% less when compared to blood-based self-testing specimens [9]. Additionally, the oral self-test was not intended to detect recent infections. It may take up to 3 months for an adequate concentration of antibodies to be detected to result in a preliminary positive test [9,10]. However, advantages of oral self-testing include privacy, less invasive route for obtaining specimen, overall test accuracy, and perceived need [9,10,11,12]. The purpose of in-home testing was to increase access to HIV self-testing outside of the clinical setting [8].

In 2019, 77 countries, including the United States, had adopted an HIV self-testing strategy [13]. HIV prevention studies have identified that in-home HIV testing is preferred among individuals because of the accessibility, privacy, and rapid results timeframe [9,10,11,12]. In-home testing, although non-traditional, supports the HIV care continuum and is vital for prevention, serostatus-awareness, and viral load suppression [14]. Despite the benefits of in-home testing, there are many challenges and barriers to use [11,15], such as comprehension of instructions and correct test usage. Among emerging adult sexual minority men, barriers to HIV testing have included low perception of vulnerability to HIV infection and the lack of access to culturally appropriate health services inclusive of LGBTQ+ populations [16,17]. Research with patients at an HIV clinic identified the unanticipated literacy and comprehension needs in understanding a health information exchange consent form [18]. Similarly, in Ghana, caregivers of children living with HIV had difficulties comprehending Westernized Likert-type levels of agreement [19]. Other extant HIV literature has described challenges related to understanding the instructions in the HIV in-home testing package insert [12,20,21,22], which has led to incorrect swabbing techniques [20,22,23], and challenges with interpretation of results [22,23]. Low and varying levels of individual health literacy can create barriers to comprehension of health-related information [24,25]. This subsequently can result in poorer health outcomes because an individual cannot understand how to appropriately address or care for their health issues [3,25]. This is compounded by the availability of health-related materials that are written using complex language, failing to clearly convey the intended health information [3,20,24,25]. HIV self-testing is a suitable, user-controlled approach to detection of new HIV cases. It can decrease the stigma associated with intentional avoidance of health care [11,20,26]. It also has the potential to facilitate awareness of HIV status among sexual minority men, especially if it is used correctly and is accessible to those who have never been tested or test infrequently [27,28]. Prior research has recommended that supplemental adaptations and labeling are needed that correspond with existing oral and blood HIV self-testing instructions in order for the test to be conducted with higher accuracy [20,22], and the utilization of pictures to convey complex information can be meaningful for health communication [29].

Our study objectives intended to address three challenges to HIV self-testing, which are: (1) correct usage of the test stick for gum swabbing, (2) understanding the number of minutes to wait before reading the result, and (3) how to correctly interpret a negative or a positive HIV result. The purpose of this study was use participatory design to create and then test the usability and comprehension of an HIV self-testing infographic in a sample of emerging adult, sexual minority men of color.

## 2. Materials and Methods

### 2.1. Design

The HIV Oral Testing Infographic Experiment (HOTIE) study was a sequential, mixed methods, randomized control trial (RCT). The study consisted of two phases in which data were collected from 2017 to 2019. The first phase consisted of participatory design sessions for HIV self-testing infographic development. During Phase 1, we convened a leadership group of 12 HIV community experts consisting of professionals in: programing for housing insecure persons, treatment and adherence evaluation, public health, health communications, community research, government programs, federal and state HIV advocacy, data science, HIV counseling and navigation, and HIV prevention. This study leveraged the expertise of an HIV interdisciplinary leadership group as designers of an infographic that would convey how to use an HIV-self test in six concise steps. In Phase 2, we tested the HIV self-testing infographic online with a sample of 322 sexual minority men ages 18 to 34.

### 2.2. Ethical Considerations

The study was approved by the institutional review boards at Yale University (IRB HSC# 1610018552) and New York University (IRB-FY2018-1573) and was registered on clinicaltrials.gov (NCT04061915).

### 2.3. Phase 1 Procedures: Infographic Development

We held three HIV self-testing infographic participatory design sessions. The design sessions lasted up to 90 min in duration. Design sessions began with a discussion about HIV self-testing. Five HIV self-testing kits were shared with the leadership group. They were asked to share the kits in teams of two and examine the kits included items. Next, the group was engaged in sketch work to put their ideas on paper for conveying how to use the test. The sketches were critiqued and new ideas for the overall design and messages to be conveyed were developed. The leadership group guided the full design of the infographic with minimal input from the researcher. A simplicity approach was used to address the three issues to self-testing reported by the leadership group during the design sessions, which have also been identified in the literature [13,20], such as: (1) incorrect swabbing technique [20,22,23], (2) confusion about the number of minutes needed to process the test [20,22], and (3) uncertainty about proper interpretation of a negative and a positive result [22,23].

**Figure 1:**
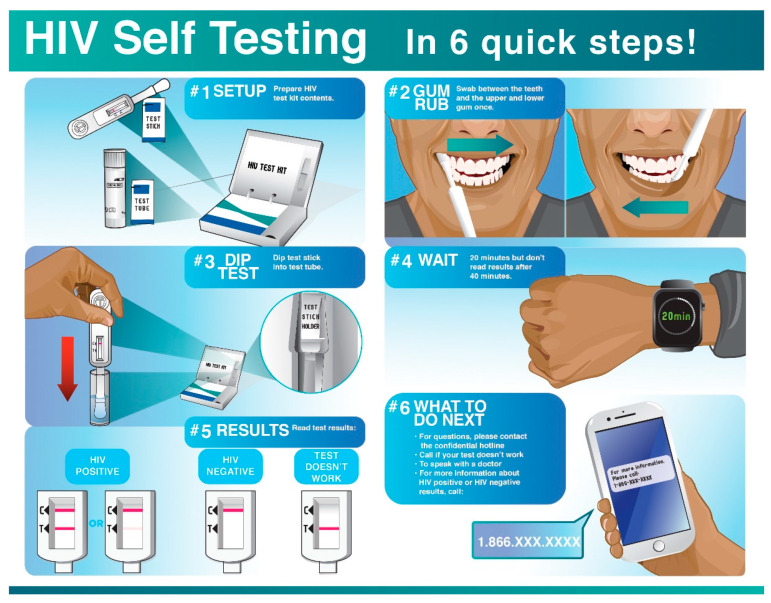
HIV self-testing infographic.

Through low-fidelity prototyping and discussion, the group identified key elements to incorporate into the infographic, such as having minimal steps from the start to completion of self-testing and minimal pieces of equipment needed to complete the test. Participants also provided input into the skin and hair color of the person represented in the infographic. Design details were very granular to the point of identifying the type of watch the person would be shown as being worn when highlighting the 20-min waiting period for the results. The last session consisted of a thorough debrief of all design iterations leading to the final infographic as well as small last-stage refinements. A graphic designer developed all iterations of the infographic, throughout Phase 1 data collection, based on the recommendations from the leadership group and oversight from the researcher. The final infographic had a total of 6 steps. The simplicity model consisted of only 3 items to be used for testing (the test stick, the developer, and the stand). Figure 1 illustrates the final iteration of the HIV self-testing infographic.

### 2.4. Phase 2 Procedures: Testing of the HIV Self-Testing Infographic Intervention

Participants were recruited online from a HIPAA-compliant, national survey panel recruitment company. The company has expertise in recruiting large samples of individuals for research and marketing panels throughout the 50 US states. This was intended to increase the reach of the intervention and mitigate risks of potential biases and imprecision that are associated with RCTs and large sample data collection. Eligibility criteria included: (1) between the ages of 18 to 34; (2) self-report as HIV-negative or unknown HIV serostatus; (3) assigned male at birth, (4) self-identify as same-gender-loving, (5) reported a sexual experience with a man in the past 12 months, and (6) able to understand and read English. Screening for eligibility occurred online. Once considered eligible, interested persons were provided information online about the purpose, risks and benefits of the study and then completed an online consent to participate or decline participation. Three hundred and twenty-two participants were randomly assigned, using automated random selection to either the intervention group (HIV self-testing infographic) or to the control group (HIV self-testing written instructions). Participants completed the intervention in one sitting, which took approximately 20 min on average. Data were collected online using a web-based survey in Qualtrics. The primary outcome was usability and comprehension of the HIV self-testing infographic. Secondary outcomes of interest included HIV knowledge, HIV self-testing, health literacy, and pre-exposure prophylaxis (PrEP) awareness.

### 2.5. Measures

Sociodemographic data collected included age (in years), race-ethnicity, education completed, employment status, individual income, current health insurance, gender, sexual attraction, HIV test results, and use of at-home HIV testing kit. We assessed gender identity using recommended questions from the World Professional Association for Transgender Health EMR Working Group (WPATH) [30]. Example questions included, “What is your current gender identity?” and “What sex were you assigned at birth on your original birth certificate?” Sexual attraction was measured by asking, “People are different in their sexual attraction to other people. Which best describes you?” [31]. HIV testing and HIV self-testing questions asked about previous testing and usage with responses being “yes or no”. Outcomes of interest included health literacy, oral HIV self-testing knowledge, HIV knowledge, oral HIV self-testing infographic usability as well as PrEP use, familiarity, and attitudes. The following instruments were used in the current study.

### 2.6. Oral HIV Self-Testing Knowledge

Oral HIV self-testing knowledge was assessed using an Oral HIV self-testing questionnaire. The Oral HIV self-testing questionnaire assessed accuracy in using oral HIV self-testing kits. Correct responses were coded as “1” and incorrect responses “0”. For the current study, responses to these five items were summed to yield a total Oral HIV self-testing knowledge score ranging from 0 to 5 (M = 3.51, SD = 1.23).

Responses were transformed into quartiles to assess the percent of questions that were responded to accurately. Recoded responses ranged “0%” (no questions responded to accurately) to “100%” accuracy (all 5 items responded to accurately), with a mean response of 70.21% (SD = 24.76). The transformed variable was highly correlated with the original combined measure (r = 0.96, *p* < 0.001), illustrating a collinear relationship.

### 2.7. Oral HIV Self-Testing Infographic Usability Questionnaire

The Oral HIV self-testing infographic usability questionnaire is a 14-item measure designed to assess usefulness and usability of the HIV oral self-testing infographic. The Oral HIV self-testing infographic usability questionnaire was derived from the Usefulness, Satisfaction, and Ease Questionnaire [32]. Responses were recorded using a 7-point Likert scale ranging from *strongly disagree* (1) to *strongly agree* (7). This measure assessed four domains that included usefulness of the HIV oral self-testing infographic (M = 5.47, SD = 1.40; Cronbach’s α = 0.92), ease of use of the HIV oral self-testing infographic (M = 5.51, SD = 1.23; Cronbach’s α = 0.85), ease of learning using the HIV oral self-testing infographic (M = 5.41, SD = 1.37; Cronbach’s α = 0.86), and satisfaction with the HIV oral self-testing infographic (M = 5.34, SD = 1.33; Cronbach’s α = 0.79). For the current study, responses to the 14-item measure were summed to yield a mean score on usability among experimental group participants only ranging from 15 to 98 (M = 5.45, SD = 1.18; Cronbach’s α = 0.95).

### 2.8. Health Literacy

The Short Assessment of Health Literacy-English, or SAHL-E is an 18-item measure designed to assess an English-speaker’s ability to read and understand common medical terms [33]. The test contains a printed common medical term, a key word (the correct response), and a distractor word. Responses were recorded dichotomously with either *false* (0) or *true* (1). Prior studies have demonstrated good internal consistency ranging from 0.80 to 0.89 [33]. For the current study, responses to 18 items were summed to yield a total score on Health literacy ranging from 0 to 18 (M = 15.47, SD = 3.53). Higher scores indicated greater health literacy (Cronbach’s α = 0.89).

### 2.9. HIV Knowledge

The Brief HIV Knowledge Questionnaire (HIV-KQ18) [34] is an 18-item *false* or *true* response measure that distinguishes understanding of HIV transmission, prevention, and consequences. Correct responses were coded as “1” with incorrect responses coded as “0”. Prior studies have demonstrated good internal consistency ranging from 0.75 to 0.89 [34]. The measure has been identified as suitable for those with low health literacy [34]. For the current study, responses to the 18 items were summed to yield a total score on HIV knowledge ranging from 0 to 18.00 (M = 11.51, SD = 4.76, Cronbach’s α = 0.84).

Responses were transformed into quartiles to assess the percent of questions that were responded to accurately. Recoded responses ranged from “0%” (no questions responded to accurately) to “100%” accuracy (all 18 items responded to accurately), with a mean response of 61.92% (SD = 27.52). The transformed variable was highly correlated with the original combined measure (r = 0.95, *p* < 0.001), illustrating a collinear relationship.

### 2.10. PrEP Use, Familiarity, and Attitudes

PrEP Use, Familiarity, and Attitudes were assessed using three separate multiple-choice questions. *PrEP use* was assessed using the question: “PrEP is the use of a medication taken before having sex as protection against HIV infection. Are you currently taking PrEP?” and measured dichotomously (0 = No, 1 = Yes; M = 0.17, SD = 0.37). *PrEP familiarity* was assessed using the question: “Truvada is a pill that HIV negative people can take to prevent HIV. This is called PrEP or Pre-Exposure Prophylaxis. How familiar are you with Truvada also called PrEP?” Responses were collected using a three-item Likert scale from 1 (*not familiar* or I do not know about PrEP) to 3 (*Familiar* or *I know about PrEP),* with a mean response of 2.22 (SD = 0.75). *PrEP attitudes* were measured using the question “How do you feel about HIV-negative people taking Truvada as PrEP to prevent transmission of HIV?” Responses were collected using a four-point Likert scale from 1 (*extremely negative*) to 4 (*extremely positive)*, with a mean response of 3.75 (SD = 0.57).

## 3. Statistical Analysis

Post hoc power analysis estimates were performed using G*Power 3.1.9 [35]. Analyses indicated that based on a type I error rate set at 0.05 and conservative effect size of 0.40 [35], and sample of 322 participants randomly assigned to experimental and control groups in this RCT study, we had 0.95 power to reject the null hypothesis with our oral HIV self-testing knowledge questionnaire.

Analyses were conducted in STATA v. 15.0. Between-group differences were examined using chi-square and independent samples t-tests to determine significant sociodemographic differences. Between-group differences using independent samples t-tests were also examined on outcomes of interest including oral HIV self-testing knowledge, HIV knowledge, and health literacy. Additional analyses were undertaken on HIV oral testing knowledge and HIV knowledge using chi-square testing to assess the percent of participants that responded accurately, allowing for more detailed understanding of participant responses. Last, linear regression analyses were conducted among experimental group participants to examine the association predictors including infographic comprehension, health literacy, PrEP Use, PrEP familiarity, and PrEP attitudes had on oral HIV self-testing knowledge and HIV knowledge. Sociodemographic variables with a *p* ≤ 0.20 were chosen as covariates for linear regression analyses to determine which sociodemographic covariates were associated with oral HIV self-testing knowledge and HIV knowledge outcomes (Bursac et al., 2008). Traditional levels such as 0.5 can fail to identify variables known to be important (Bursac et al., 2008). Covariates were retained based on meaningful contribution and statistical significance to the final analytical model (Aneshenseel, 2012). Cases with missing data were excluded from the analyses. Of the 360 participants in the parent study, 11.12% were excluded for missing data, generating a final sample of 322 participants for the current analyses. Imputation was not used to address missingness in any of the analyses.

## 4. Results

### 4.1. Between-Group Differences on Sample Socio-Demographic Characteristics

Sample sociodemographic characteristics are presented in Table 1. All participants were men sexually attracted to men. Participant ages ranged between 18 and 34 years, with the majority of participants between 25 and 34 years of age (65%). Participants were predominantly Hispanic/Latinx (49%) and African American (38.2%). All participants self-identified as persons of color. In the sample, the 22% who identified as non-Hispanic white also identified as having racial/ethnic heritage (e.g., non-Hispanic white + Asian or + African American). Participant education ranged from less than a high school education (2%) to doctoral degree (1.3%), with 38 percent having obtained a four-year college degree (29.2%) or professional degree (8.8%). A larger proportion of participants were employed full-time (52.0%) and 56.5 percent of participants having an annual income that ranged from less than $10,000 per year to $39,999 per year. Approximately 63.1 percent were either on their parent’s health insurance (16%), had private health insurance (36.4%), or state-sponsored insurance (10.7%); however, 20.7 percent had no health insurance. All participants had an HIV test in the 6 months prior to participating in the survey with 85 percent having a confirmed negative HIV test result. Majority of participants have never used an at-home self-test HIV kit (85.6%). No statistically significant differences were identified between experimental and control groups on sociodemographic characteristics.

### 4.2. Between-Group Differences on Oral HIV Testing Knowledge, HIV Knowledge, and Health Literacy

Table 2 shows the breakdown of outcomes of interest between experimental and control groups. Between-group mean-level differences were identified on oral HIV self-testing knowledge accuracy (t[318] = 3.96, *p* < 0.001). Male participants in the control group had higher mean oral HIV self-testing knowledge accuracy scores (M_oral HIV testing_ = 74.56, SD = 23.25) as compared to participants in the experimental group (M_oral HIV testing_ = 65.18, SD = 25.19), with a moderate effect size identified (*d* = 0.44). No mean-level differences were identified between groups on HIV knowledge percent accuracy between the control group (M_HIV knowledge_ = 58.76, SD = 27.95) and experimental group (M_HIV knowledge_ = 57.01, SD = 27.63). Between-group mean-level differences were identified on health literacy (t[318] =2.93, *p* = 0.004). Male participants in the experimental group had higher mean health literacy (M_Health literacy_ = 68.21, SD = 28.26) when compared to the control group (M_Health literacy_ = 59.17, SD = 26.87). A moderate effect size was identified (*d* = 0.33).

**Figure 2:**
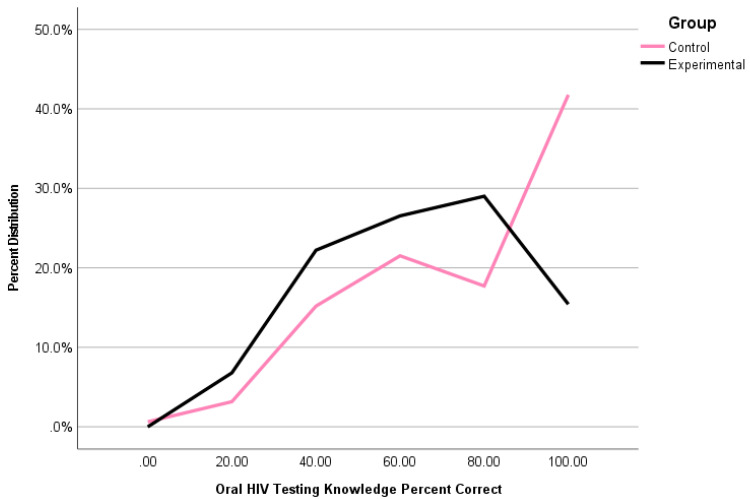
Percentage distribution of accuracy of oral HIV self-testing knowledge (in percentages) between experimental (*n* = 161) and control groups (*n* = 161).

Table 3 displays the percent accuracy on oral HIV self-testing knowledge questions and HIV knowledge questions, using chi-square testing. While the control group showed greater mean-level responses in oral HIV self-testing knowledge accuracy (see Table 2), a significantly greater percentage of participants in the experimental group showed up to 80% accuracy (χ*^2^* = 5.68, *p* = 0.02) in responses, when compared to the control group; however, a statistically significant (χ*^2^* = 27.27, *p* < 0.001) and larger proportion of participants showed 100% accuracy in the control. See Figure 2. HIV knowledge showed no variation based on chi-square difference testing (χ*^2^* = 1.94, *p* = 0.75).

### 4.3. Feasibility and Acceptability of the Infographic and Exploratory Analyses of Predictors of Oral HIV Testing Knowledge and HIV Knowledge among Experimental Group Participants

**Figure 3:**
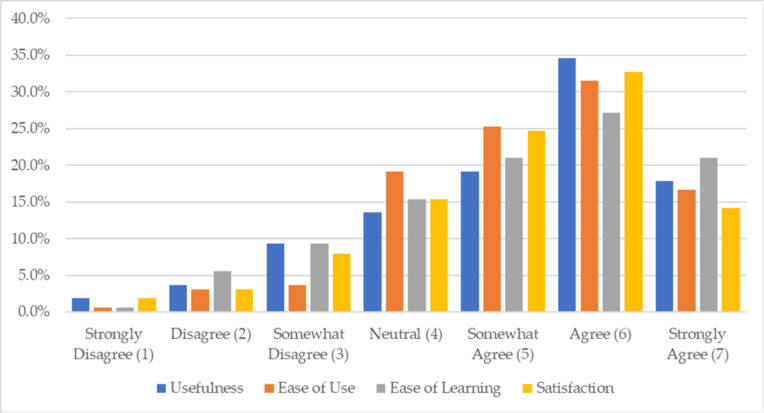
Bar chart display of experimental group participant responses on the feasibility and acceptability on the oral HIV self-testing infographic questionnaire (*n* = 161).

Figure 3 and Table 4 display the response rate for each of the four domains on feasibility and acceptability of the infographic among experimental group participants based on response options ranging from *Strongly Disagree* (1) to *Strongly Agree* (7). Over 50 percent of participants “somewhat agreed” to “strongly agreed” it was useful, easy to use, felt it was easy to learn, and were generally satisfied with the infographic. Participant mean responses showed “somewhat” agreeance on the infographic usefulness (M = 5.47, SD = 1.40), infographic ease of use (M = 5.51, SD = 1.23), infographic ease of learning (M = 5.41, SD = 1.37), and satisfaction with the infographic (M = 5.34, SD = 1.33). Ease of learning and satisfaction with the infographic had the lowest mean responses.

Correlation analyses were conducted among the four domains on feasibility and acceptability of the infographic with oral HIV self-testing knowledge, HIV knowledge, and PrEP Use, PrEP familiarity, and PrEP attitudes (see Table 5). Results indicate that the four domains on feasibility and acceptability of the infographic were all highly correlated (*p* < 0.001). These domains on feasibility and acceptability of the infographic were also each correlated with oral HIV self-testing knowledge and HIV knowledge (*p* < 0.001). Interestingly, the four domains on feasibility and acceptability of the infographic were not correlated with health literacy, PrEP Use, PrEP familiarity, and PrEP attitudes, as predictors of oral HIV self-testing knowledge and HIV knowledge. Oral HIV self-testing knowledge was positively correlated with health literacy, PrEP use and PrEP attitudes (*p* < 0.05). HIV knowledge was positively correlated with health literacy and PrEP familiarity (*p* < 0.05). Given the pattern of correlations among these variables, exploratory linear regression analyses were conducted to further assess the association the feasibility and acceptability of the infographic, health literacy, PrEP Use, PrEP familiarity, and PrEP attitudes had with oral HIV self-testing knowledge and HIV knowledge.

Table 6 presents findings from linear regression analyses among experimental group participants (*n* = 161). Infographic feasibility and acceptability, health literacy, PrEP Use, PrEP familiarity, and PrEP attitudes were examined as indicators of oral HIV self-testing knowledge and HIV knowledge. Sexual attraction, highest level of education, and prior use of at-home HIV testing kit were examined for inclusion in regression analyses because of the significance between groups, or with a *p* ≤ 0.20 (see Table 1) [36]. Traditional levels such as 0.5 can fail to identify variables known to be important [36]. Highest level of education was retained in final model due to statistically significant contribution [37].

Linear regression analyses showed that infographic comprehension was significantly associated with oral HIV self-testing knowledge (β = 0.33, *t* = 4.50, *p* < 0.001) and HIV knowledge (β = 0.19, *t* = 2.85, *p* = 0.005). Health literacy, while not associated with oral HIV self-testing knowledge (β = 0.09, *t* = 1.08, *p* = 0.28), was significantly associated with HIV knowledge (β = 0.32, *t* = 4.55, *p* < 0.001). PrEP use (β = 0.15, *t* = 2.00, *p* = 0.04), PrEP familiarity (β = 0.18, *t* = 2.18, *p* = 0.03), and PrEP attitudes (β = 0.15, *t* = 1.92, *p* = 0.05) were all significantly associated with oral HIV self-testing knowledge. PrEP familiarity was associated with HIV knowledge (β = 0.19, *t* = 2.52, *p* = 0.01). These models accounted for a significant proportion of the variance in oral HIV self-testing knowledge (R^2^= 0.25, F (6) = 8.87, *p* < 0.001) and HIV knowledge (R^2^= 0.26, F (6) = 11.96, *p* < 0.001).

## 5. Discussion

The purpose of this study was to design and test the acceptability, feasibility, and comprehension of an HIV self-testing infographic in a sample of 322 emerging adult sexual minority men ages 18–34. Participatory design was used to create a researcher, co-design, iterative process for a tailored and comprehensible HIV oral, self-testing infographic [2]. Participatory design is not carried out using a strict set of rules or formulas [38], but rather with a commitment to ongoing participation, with the researcher giving limited input as a co-designer [38].

The Phase 1 leadership group provided thoughtful insights and professional expertise on all aspects of the infographic (simple wording, minimal items for testing, etc.), following the advice of Peck et al.,2014 to minimize the scope for error in self-testing by minimizing the number of test components to enhance usability [22]. The leadership group was also able to help tailor the infographic to emphasize concepts like “waiting 20 min” and that a “faint line” is still a positive test. These adjustments elevated the infographic. Moreover, the leadership group conveyed issues presented by a “faint line” on the test stick where persons were unable to decipher a negative and a preliminary positive result for user education. Lastly, because of the leadership group’s feedback small, brief wording was incorporated into the infographic to decrease confusion. Tailored infographics, similar to other HIV prevention studies using multimedia messaging and tailored approaches, can facilitate steps towards behavioral change [39,40].

We found no significant differences among racial-ethnic groups and socio-demographic characteristics. In the intervention, all participants in the experimental and control groups received information on how to use an HIV self-test. Seventy percent of the sample reported the infographic was useful; 73% reported it was easy to use, and 69% agreed at some level that the infographic was easy to learn about HIV self-testing.

Our results revealed a significant mean difference between the control and experimental groups with respect to HIV self-testing knowledge. The control group was administered written instructions on HIV self-testing. Control group participants had higher mean oral HIV self-testing knowledge accuracy scores, suggesting that more participants in the control group got all the questions about HIV Oral Self Testing correct when compared to the experimental group. We surmise that this was because approximately 76% of the sample had some college education or higher, and that may have led to their outperforming the intervention group. Findings for the infographic are similar to other studies using a visual aid for HIV-related decision making [41,42], which suggests that the benefits of using an infographic can be best optimized when combined with a dedicated person to reinforce information.

However overall, both treatment and control groups performed well on three significant areas identified in the literature as a challenge to HIV self-testing. First, both groups demonstrated understanding that they had to wait 20 min until the result appeared (67% accuracy intervention group; 76% accuracy control). Second, participants demonstrated understanding that the swabbing had to cover both the upper and lower gum lines (69% accuracy intervention group; 82% accuracy control). Last, participants demonstrated understanding that a pink line in both the control and the test, even if the line was faint, should be interpreted as a preliminary positive result (62% accuracy intervention group; 72% accuracy control). The infographic was still somewhat successful at facilitating comprehension of the three critical testing steps that have been identified as challenges in other studies.

For example, a study testing the feasibility and acceptability of oral and blood specimen HIV self-testing in Kenya, Malawi, and South Africa found that of the subgroup of participants (*n* = 33) who used the oral testing, 51% waited the correct number of minutes before reading the test result, less than 40% obtained the oral specimen (oral swabbing) correctly, and almost 40% conducted all steps correctly [22]. Additionally, most of the participants in that study who used the oral test believed that the self-test was acceptable to use [22], which is consistent with other research on the use of in-home testing [26,43,44,45]. Despite the high acceptability of HIV oral self-testing [46,47,48], there can still be some inaccuracies with individual administration of the test [22,46,47] and reluctance to access testing [49,50].

The majority of our sample identified having health insurance and had been tested for HIV in the past. Only 14% of participants reported ever having used an HIV oral self-test. Additionally, results from our linear regression suggested that PrEP attitudes and use were positively associated with HIV self-testing knowledge, which is consistent with studies in this area [51]. HIV self-testing can be used in persons already taking antiretroviral therapy to supplement clinic-based testing and encourage routine or more frequent testing [52]. Interventions that optimize HIV self-testing to identify new cases through consistent tests can promote linkage to care and biomedical prevention for HIV negative individuals, such as PrEP and post-exposure prophylaxis (PEP). Innovative approaches are needed to improve HIV-self testing among populations that are vulnerable to HIV infection.

### 5.1. Participatory Design for HIV Prevention

The overall findings from this study suggested that the infographic was somewhat successful. Upwards of two-thirds or better of the participants in the intervention group were able to comprehend the three critical steps for accurately using an HIV self-test. Our approach was guided by recommendations for participatory design [2,3], which added methodological rigor to the design phase. The HIV leadership group was interdisciplinary, and their expertise in HIV prevention in sexual minority populations provided a diversity of ideas for infographic design. The researchers had limited input into the design to minimize bias and allowed the leadership group to lead using their tacit knowledge. The design sessions included exploration of physical HIV self-testing kits, low-fidelity prototyping, and interactive discussions.

### 5.2. Recommendations for Future Participatory Design Studies Using Visual Aids for HIV Prevention

We have several recommendations, based on our study findings, for future participatory design approaches for HIV prevention. First, a peer advisory board from the target study population should be established. These individuals should be involved with the participatory design leadership group on all aspects of design and implementation. However, the peer advisory board will be unable to participate in the intervention because any in-depth knowledge acquired from participating in the design would decrease the validity of the interventions study findings. Second, the peer advisory board should be compensated for their time and expertise based on their lived experience. Establishing collaborations of this nature should not be for one-time data collection. This is a relationship that should be valued and cultivated over time. Researchers should invest in establishing trustworthiness to build trust [53,54]. Third, the peer advisory board should not only provide expertise in the design phases. They should also be included in study implementation. Strong visualization designs, informed by a peer advisory board and in consort with a leadership group, may not perform as expected if the intervention is not administered in a culturally informed manner, such as acknowledging time, location, technological modality to be used, and community gatekeepers. Fourth, the peer advisory board should be offered the opportunity to be included in manuscript authorship. Highly important to the success of the study is expert guidance provided in the early developmental stages. Extending the opportunity for the peer advisory board to be a part of dissemination activities, such as authorship, would demonstrate an appreciation of their valuable and unique contributions.

### 5.3. Limitations

Several limitations were noted. First, self-reported data are subject to recall and social desirability bias. There is a possibility that participants may underreport responses to sensitive HIV, PrEP, or literacy questions. Because we used online data collection, the risk of this may have been minimized. Second, generalizability of our findings to the larger community of emerging adult sexual minority men is limited due to sample size. However, having a sample represented from all US regions with diverse ethnic and racial representation along with the study findings, provide unique insights into understanding HIV prevention approaches and considerations for literacy and comprehension needs. Third, cross sectional data were collected on PrEP attitudes, HIV Knowledge, and HIV testing. Longitudinal data would be more advantageous to examine knowledge and behaviors over time. Fourth, the control group outperformed the intervention group. We believe this was due to not distributing certain characteristics, such as level of education, evenly between the intervention and control groups during randomization. To prevent this from occurring in future intervention work, one option would be to use stratified randomization. Last, although participatory design is a rigorous approach for tailored design of a tool for a behavioral intervention, it may not always be entirely successful. Inclusion of a peer advisory board, comprised of the target population for the intervention, may have improved infographic design and comprehensiveness.

## 6. Conclusions

The purpose of this study was to design and test an HIV self-testing infographic in emerging adult sexual minority men. Based on our findings, the intervention group did not perform as well as the control group. However, more than 60% of the intervention group were able to comprehend the three critical steps identified in the literature that have been challenges to accurately using the self-test and interpreting the results. This was a promising finding that has resulted in the authors’ development of additional recommendations for using participatory design in visual aid development for HIV prevention. HIV self-testing is advantageous for HIV prevention. It allows for private, rapid testing, and may help reduce stigma, which has been to be a major barrier. Participatory design of an HIV self-testing infographic is a rigorous approach as a health communication strategy to address public health priorities.

## Figures and Tables

**Table 1:** Between-group differences on sociodemographic characteristics.

Sociodemographic Characteristic	Total Sample (*n* = 322)	Experimental Group (*n* = 161)	Control Group (*n* = 161)	Test Statistic (t, χ^2^)	*p*-Value
**Age**				0.21	0.65
18 to 24 years	114 (35.0%)	59 (51.8%)	55 (48.2%)		
25 to 34 years	208 (65.0%)	102 (49.0%)	106 (51.0%)		
**Gender**				--	--
Male	322 (100%)	161 (100%)	161 (100%)		
**Race-Ethnicity ^a^**					
Hispanic/Latinx Identity	157 (48.9%)	79 (48.8%)	78 (49.0%)	0.01	0.91
Black/African American Identity	122 (38.2%)	60 (37.0%)	62 (39.5%)	0.16	0.68
Asian Identity	69 (21.6%)	36 (22.2%)	33 (21.0%)	0.08	0.77
White non-Hispanic Identity	74 (22.6%)	36 (22.2%)	38 (22.9%)	0.06	0.79
American Indian/Native American Identity	17 (5.6%)	10 (6.8%)	7 (4.5%)	0.83	0.36
Middle Eastern Identity	10 (3.1%)	7 (4.3%)	3 (1.9%)	1.55	0.21
**Education**				4.17	0.15
Less than high school	5 (1.6%)	2 (1.2%)	3 (1.9%)		
High school graduate/GED	77 (23.8%)	37 (22.8%)	40 (24.8%)		
Some College	75 (23.2%)	34 (21.0%)	41 (25.5%)		
2-year degree	38 (12.2%)	18 (11.7%)	20 (12.7%)		
4-year degree	95 (29.2%)	49 (30.2%)	46 (28.0%)		
Professional degree	28 (8.8%)	18 (11.1%)	10 (6.4%)		
Doctorate	4 (1.3%)	3 (1.9%)	1 (0.6%)		
**Employment Status**				1.45	0.96
Employed Full-time	167 (52.0%)	84 (51.9%)	83 (52.2%)		
Employed Part-time	54 (16.9%)	28 (17.3%)	26 (16.6%)		
Self-employed	19 (5.95%)	9 (5.6%)	10 (6.4%)		
Unemployed looking for work	30 (9.1%)	15 (9.3%)	15 (8.9%)		
Unemployed not looking for work	4 (1.3%)	3 (1.9%)	1 (0.6%)		
Student	45 (13.8%)	23 (13.6%)	22 (14.0%)		
Disabled	3 (.9%)	1 (0.6%)	2 (1.3%)		
**Income**				11.56	0.39
Less than $10,000	58 (17.9%)	34 (21.1%)	24 (14.6%)		
$10,000–$19,999	35 (11.3%)	16 (10.6%)	19 (12.1%)		
$20,000–$29,999	49 (15.4%)	19 (11.8%)	30 (19.1%)		
$30,000–$39,999	38 (11.9%)	20 (12.4%)	18 (11.5%)		
$40,000–$49,999	31 (9.7%)	15 (9.3%)	16 (10.2%)		
$50,000–$59,999	24 (7.5%)	10 (6.2%)	14 (8.9%)		
$60, 000–$69,999	20 (6.3%	9 (5.6%)	11 (7.0%)		
$70,000–$79,999	19 (5.7%)	9 (5.6%)	10 (5.7%)		
$80,000–$89,999	10 (3.1%)	4 (2.5%)	6 (3.8%)		
$90,000–$99,999	14 (3.8%)	8 (5.0%)	6 (3.8%)		
$100,000–$149,000	12 (3.8%)	7 (4.3%)	5 (3.2%)		
More than $150,000	11 (3.5%)	9 (5.6%)	2 (1.3%)		
**Health Insurance**				4.06	0.54
Parent’s Health Insurance	51 (16.0 %)	28 (17.9%)	23 (14.0%)		
Private health Insurance	117 (36.4%)	53 (32.7%)	64 (40.1%		
State Sponsored Health Plan	35 (10.7%)	16 (9.9%)	19 (11.5%)		
Medicaid	43 (13.0%)	26 (16.0%)	17 (10.8%)		
Military Health Care (TRICARE/VA/CHAMP–VA)	10 (2.8%)	5 (3.1%)	5 (2.5%)		
No Health Insurance	66 (20.7%)	33 (20.4%)	33 (21.0%)		
**HIV Test Results**					
Negative Result	274 (85.0%)	135 (84.0%)	139 (86.0%)		
Unclear Result	10 (3.1%)	5 (3.1%)	5 (3.2%)		
I have not received my test results	38 (11.9%)	21 (13.0%)	17 (10.8%)		
**Used an at-home HIV Testing Kit**				1.40	0.20
Yes	46 (14.4%)	27 (16.7%)	19 (12.1%)		
No	276 (85.6%)	134 (83.3%)	142 (87.9%)		

^a^ Separate responses recorded for race-ethnicity.

**Table 2:** Outcomes of interest between experimental group and control group (*n* = 322).

	Experimental Group (*n* = 161)	Control Group (*n* = 161)	t (320)	*p*-Value	Cohen’s d	CI 95%
Oral HIV Self-Testing Knowledge ^a^	65.18 (25.19)	74.56 (23.25)	3.96	<0.001	−0.44	[−0.66, −0.22]
HIV knowledge ^a^	57.01 (27.63)	58.76 (27.95)	0.31	0.76	0.03	[−0.18, 0.25]
Health literacy	68.21 (28.26)	59.17 (26.87)	−2.93	0.004	0.33	[0.11, 0.55]

^a^ Reflect the percent accuracy on oral HIV self-testing knowledge and HIV knowledge questions.

**Table 3:** Chi-square test on oral HIV self-testing knowledge and HIV knowledge accuracy between experimental group and control group.

	Experimental Group (*n* = 161)	Control Group (*n* = 161)	χ^2^	*df*	*p*-Value
**Oral HIV Self-Testing Knowledge Accuracy**			29.94	5	<0.001
0%	0 (0.0%)	1 (0.6%)	1.03	1	0.31
20%	11 (6.8%)	5 (3.2%)	2.21	1	0.13
40%	36 (22.2%)	24 (15.2%)	2.59	1	0.11
60%	43 (26.5%)	35 (21.5%)	1.11	1	0.29
80%	46 (29.0%)	29 (17.7%)	5.68	1	0.02
100%	25 (15.4%)	67 (41.8%)	27.27	1	<0.001
**HIV Knowledge Accuracy**			1.94	4	0.75
0%	7 (0.04%)	3 (0.02%)	1.55	1	0.21
25%	36 (22.3%)	35 (21.7%)	0.02	1	0.98
50%	43 (26.7%)	40 (24.8%)	0.06	1	0.80
75%	40 (24.8%)	43 (26.7%)	0.07	1	0.79
100%	35 (21.7%)	40 (24.8%)	0.53	1	0.51

**Table 4:** Experimental group participant responses on the feasibility and acceptability on the oral HIV self-testing infographic questionnaire (*n* = 161).

Response	Usefulness	Ease of Use	Ease of Learning	Satisfaction
Strongly Disagree (1)	1.9%	0.60%	0.60%	1.90%
Disagree (2)	3.7%	3.10%	5.60%	3.10%
Somewhat Disagree (3)	9.3%	3.70%	9.30%	8.00%
Neutral (4)	13.6%	19.10%	15.40%	15.40%
Somewhat Agree (5)	19.1%	25.30%	21.00%	24.70%
Agree (6)	34.6%	31.50%	27.20%	32.70%
Strongly Agree (7)	17.9%	16.70%	21.00%	14.20%

**Table 5:** Correlation matrix of main analytic measures among experimental group participants (*n* = 161).

	1	2	3	4	5	6	7	8	9	10
1. Infographic Usefulness	1	0.71 **	0.64 **	0.80 **	0.31 **	0.18 **	0.03	0.01	−0.03	0.13
2. Infographic Ease of Use		1	0.78 **	0.68 **	0.35 **	0.20 **	0.06	−0.04	−0.05	−0.01
3. Infographic Ease of Learning			1	0.71 **	0.39 **	0.25 **	0.03	−0.03	−0.01	−0.01
4. Satisfaction with the infographic				1	0.27 **	0.25 **	0.03	−0.03	−0.02	0.06
5. Oral HIV self-testing knowledge					1	0.16 *	0.16 *	0.17 *	−0.12	0.16 *
6. HIV knowledge						1	0.37 **	−0.12	0.30 **	0.06
7. Health Literacy							1	−0.14	0.08	0.14
8. PreP Use								1	0.25 **	0.13
9. PrEP Familiarity									1	0.27 **
10. PrEP Attitudes										1

** *p* < 0.01, * *p* < 0.05.

**Table 6:** Linear regression on oral HIV self-testing knowledge percent accuracy and HIV knowledge percent accuracy among experimental group (*n* = 161).

	Oral HIV Self-Testing Knowledge				HIV Knowledge			
	β	SE	*t*	*p*-Value	β	SE	*t*	*p*-Value
Feasibility and acceptability of the infographic	0.33	0.10	4.50	<0.001	0.19	0.02	2.85	0.005
Health Literacy	0.09	0.65	1.08	0.28	0.32	0.01	4.55	<0.001
PreP Use ^a^	0.15	0.45	2.00	0.04	−0.13	0.90	−1.83	0.19
PrEP Familiarity	0.18	0.55	2.18	0.03	0.19	0.48	2.52	0.01
PrEP Attitudes	0.15	0.91	1.92	0.05	0.013	0.38	0.17	0.86
Highest Level of Education	0.29	0.13	2.76	0.006	0.18	0.23	2.68	0.008
F (*df*)	8.87 (6)			<0.001	11.96 (6)			<0.001
*R* ^2^	0.25				0.26			

Note: PrEP = Pre-Exposure Prophylaxis; SE = Standard Error. ^a^ 0 = No, 1 = Yes.

## Data Availability

The datasets generated during and/or analyzed during the current study are not publicly available, but are available from the corresponding author on reasonable request.
